# Arts Therapies Interventions and Their Outcomes in the Treatment of Eating Disorders: Scoping Review Protocol

**DOI:** 10.3390/bs10120188

**Published:** 2020-12-09

**Authors:** Monika Bucharová, Andrea Malá, Jiří Kantor, Zuzana Svobodová

**Affiliations:** 1 Palacky University Evidence-Based Education Working Team: Mentee Centre, and Institute of Special Education Studies, Faculty of Education, Palacky University, 779 00 Olomouc, Czech Republic; maladmp@gmail.com (A.M.); jiri.kantor@upol.cz (J.K.); 2 Czech National Centre for Evidence-Based Healthcare and Knowledge Translation (Cochrane Czech Republic, Czech Evidence-Based Healthcare Centre: Joanna Briggs Institute Centre of Excellence, Masaryk University GRADE Centre), Institute of Biostatistics and Analyses, Faculty of Medicine, Masaryk University, Kamenice 3, 625 00 Brno, Czech Republic; zuzana.kelnarova@gmail.com

**Keywords:** arts therapies, music therapy, art therapy, drama therapy, dance/movement therapy, dance movement psychotherapy, dance therapy, eating disorder, anorexia, bulimia

## Abstract

Arts therapies (AsTs) are considered a valuable intervention for people with eating disorders, however the range of research studies and the comparison between the types of arts therapies are unknown. The goal of the future scoping review is to explore the therapeutic outcomes addressed by arts therapists in research studies on people with eating disorders and compare the different types of arts-based interventions. This scoping review will be conducted in accordance with the Joanna Briggs Institute methodology. Included will be research studies and sources oriented towards people with eating disorders of all ages and AsTs of any type (art therapy, drama therapy, music therapy, dance/movement therapy, and expressive therapies). There is no language/publication period limitation. The following databases will be searched: CINAHL Plus, EMBASE, MEDLINE (OvidSP), ProQuest Central, PsycINFO, PubMed, Scopus, and Web of Science. Sources of unpublished studies and grey literature will include Google Scholar, MedNar, clinical trials, and current controlled trials. Titles/abstracts and full texts of studies will be assessed against the inclusion criteria, and the data extracted by two independent reviewers. Based on the results, we will compare the types of AsTs according to the research designs, country/settings, intervention methods/materials, adverse effects reported, and therapeutic outcomes related to AsTs.

## 1. Introduction

Eating disorders (EDs) are mental illnesses that cause serious disturbances to people’s everyday diet. Since EDs are on the rise throughout the world, healthcare policies and practitioners need to research different treatment options and their affectivity, value for patients, and cost effectiveness, in order to make any substantial change to the growth of this disease. The goal of this paper is to explore the contribution arts therapies (AsTs) bring to the treatment of patients with EDs, based on the analysis of AsTs interventions and possible outcomes of the treatment. In the introduction section of this protocol, the theoretical framework including different aspects of EDs; their prevalence, definition, and treatment, plus the unique features of different AsTs modalities, will be presented, followed by the justification and objectives of this review proposal.

### 1.1. Eating Disorders and Their Clinical Aspects

Once seen as a westernized condition, the shift of EDs into the global population is accelerating [1,2,3]. Erskine et al. [4] states that the inclusion of EDs in the Global Burden of Disease Study (GBD) is a milestone in the recognition of the wider health issue affecting the global community. The Smink et al. [5] review of the worldwide epidemiology of eating disorders states that anorexia nervosa (AN), bulimia nervosa (BN), and binge eating disorder (BED), combined, ranked as the 12th leading cause of disability-adjusted life years (DALYs) in females between the ages of 15–19. Epidemiological studies show a clear increase in the prevalence of EDs over a study period, from 3.5% for the 2000–2006 period to 7.8% for the 2013–2018 period, which seems alarming [6]. According to the National Eating Disorder Association (NEDA) [7] up to 30 million people in the United States alone suffer from an ED. Furthermore, Qian et al. [8] found that EDs are more common in Western countries compared to Asian countries [8].

Currently there are many discussions as to why we are experiencing such a growth in ED cases. Grave [9] suggests there are various and combined causal factors such as psychological, sociocultural, biological, or family factors. People of all ages and genders, as well as cultural backgrounds, can be affected by an eating disorder [10]. On one hand, experts are partly attributing the increase to a greater awareness of the wide range of disorders and the changes in diagnostic criteria [8]. On the other hand, shifts in populations and cultural influences, the advances in technology with the surge in social media and the focus on body shape and gender roles, combined with the questioning of traditional family and work structures are contributing to changes in what is seen as healthy eating and a healthy body image.

EDs can manifest as eating extremely small amounts of food or severely overeating. The National Health Service (NHS) [11] terms EDs as an unhealthy attitude to food; an attitude by which the focal point of a person´s life is their relationship to food. In addition to maladaptive eating patterns are concerns, even obsessions, around weight and body shape/image. These behaviors are classified as mental health illnesses not because of the significant impact on the body’s ability to get appropriate nutrition, but because of the psychological distress, fear of gaining weight, distorted body image, and excessive exercise, which then categorizes the ED as a biologically-influenced medical illness [12]. Further evidence shows that eating behaviors are affected by emotional regulation. Specifically, higher levels of alexithymia, a personality trait characterized by an inability to modulate and identify one’s feelings and body sensations, were found in overweight persons [13]. Alexithymia frequently co-occurs with depression, anxiety, social phobia, and substance abuse, all of which are disorders which often overlap with an ED.

Eating disorders have their own specific diagnostic criteria according to both major diagnostic manuals. The American Psychiatric Association’s fifth edition of Diagnostic and Statistical Manual of Mental Disorders (DSM-5) provides diagnostic criteria for pica, rumination syndrome, avoidant/restrictive food intake disorder, AN, BN, and BED [14]. The tenth revision of the International Statistical Classification of Diseases and Related Health Problems (ICD-10) by the World Health Organization provides slightly different diagnostic criteria for EDs [15]. ICD-10 recognizes diagnostic criteria for AN, atypical AN, BN, atypical BN, overeating associated with other psychological disturbances, vomiting associated with other psychological disturbances, and other eating disorders. It is common to observe migration between ED diagnoses as well as frequent use of unspecified eating disorder diagnoses [9], however, AN and BN are known as the most predominant eating disorders.

Studies point out that EDs have, in recent years, had the highest mortality risk among other psychiatric diagnoses [5], however the exact mechanism for how a person’s susceptibility to an ED might work is not fully understood. Research in general concurs that a combination of genetic, biological, behavioral, psychological, and social factors can raise a person’s risk [9]. Although an ED can be developed at any age, the National Institute for Health and Care Excellence (NICE) reminds us that the highest risk of development is seen in young women and men between 13 and 17 years of age [2]. Certain psychological factors and personality traits which develop during these adolescent years may predispose a person to an eating disorder [14]. Neuroticism, obsessiveness, and perfectionism can play a large role in facilitating EDs, particularly AN and BN. Individuals with these personality features are prone to anxiety and depression, and display perfectionistic and self-critical tendencies, all of which are factors that may contribute to their difficulty managing their weight and eating in a healthy manner [7,14]. Fassino et al. [16], in their controlled study of the temperament and personality of eating disorder patients, state that those diagnosed with AN tend to have high levels of harm avoidance, characterized by worrying and pessimism, and low levels of novelty seeking, characterized by a rigidity in thinking and an unwillingness towards new experiences. Individuals diagnosed with BN also show high levels of harm avoidance, but instead is it combined with high levels of novelty seeking, which is linked to the lack of impulse control associated with binge–purge behaviors [16].

For some years now it has been suggested that a developmental understanding of disordered eating would be greatly enhanced if we were to look through the lens of developmental psychopathology, more specifically the clarity and distortions that emerge from primary relationships [17]. An insecure attachment is the key risk factor to developing an ED. Attachment theory, originating from the work of Bowlby [18], offers a comprehensive framework for understanding the potential individual and family characteristics which contribute to the development of an ED. Furthermore, it provides an insight into a range of different psychological functions like emotion regulation and interpersonal functioning, which are associated with EDs [19]. Mantillia et al. [20] and Gander et al. [19] extend this point by arguing that the relationship a person has with their eating disorder is shaped by that person’s understanding of what meaningful relationships should look like, this in turn has important consequences on the severity of their disorder.

Mantillia et al. [20] uses “attachment theory” to establish a link between the attachment to a significant other and the attachment to an ED. The study group researched consisted of 148 women with EDs aged from 16–25 in an outpatient setting in Sweden. ED behaviors were measured by questions looking at the regularity and the relationship of the ED to the patient. The findings showed that the less securely attached individuals experienced their ED as more in control, and themselves as less autonomous [20]. Findings from Gander et al.’s [19] study of research and literature that used a narrative-based methodology in the field of EDs in adolescences, showed a high prevalence of unresolved attachment status caused by possible abuse. So instead of choosing to be close to someone consistently, this adult attachment behavior in the patient escapes consistently into the ED in order to overcome the emotional/physical injury. A plausible link can therefore be made from this data which suggests that insecure and unresolved (disorganized) attachment types produce a tendency to internalize unexpressed emotions and the expectation of a controlling relationship which in turn produces disordered eating symptoms as a means to survive and manage the unhealthy relationship.

The treatment of EDs is one of the most difficult among other psychiatric disorders. Currently there is a large spectrum of therapeutic approaches and a strong therapeutic alliance between therapist and patient is emphasized [21]. Various forms of cognitive behavioral therapy (CBT) are widely used [21,22]. Enhanced cognitive behavioral therapy (CBT-E) was specifically developed to maintain eating disorder psychopathology and studies support its efficacy and effectiveness [23,24]. Another approach offers cognitive remediation therapy (CRT) which focuses on core cognitive processes [25]. Some of the newer approaches in the treatment of EDs are the Maudsley Anorexia Nervosa Treatment for Adults (MANTRA), as well as specialist supportive clinical management (SSCM). SSCM combines features of clinical management and supportive psychotherapy [26]. After unsuccessful application of CBT, MANTRA, or SSCM, eating-disorder-focused focal psychodynamic therapy (FPT) is applied [2]. A family-based approach for children and young people with EDs which shows promising results especially at follow-up care, and according to given evidence may be more beneficial than individual therapy [25,27]. Medication should not be offered as a sole treatment for AN, BN, or BED [2]. However pharmacotherapy is frequently prescribed in combination with CBT or other psychotherapy as a means to help decrease other psychological symptoms such as anxiety or depression [25,28]. At this stage it is challenging to describe the efficacy of each treatment approach. Outcomes of residential care in various studies reported positive results of in-patient treatment, however the difficulty remains in identifying which approach provides the most efficient outcomes since each program combines them differently [29].

### 1.2. Arts Therapies and Their Modalities in the Treatment of Eating Disorders

Arts therapy (AsT) disciplines which have been developed as the result of multidisciplinary efforts between artists, psychotherapists, educators, and social/health workers share common characteristics, namely the value they each place upon creativity. They each share an appreciation of the non-verbal aspect of communication and understand the use of imagery, symbolism, and metaphor as a link to psychological/emotional states. They acknowledge the need to work safely in the presence of a secure therapeutic relationship, guided with interventions that are based on the therapeutic aims of the specific individual as well as the client population [30].

A central premise of the AsTs is that any individual regardless of ability, disability, illness, or health can engage creatively in the arts and use them to help restore health and well-being. It is this open position to engage creatively that gives AsTs a unique contribution to the treatment of EDs. AsTs (also creative therapies (CTs), or creative arts therapies (CAsTs)) are defined as “the creative use of the artistic media (visual art, music, drama, and dance/movement) as vehicles for non-verbal and/or symbolic communication, within a holding environment, encouraged by a well-defined client–therapist relationship, in order to achieve personal and/or social therapeutic goals appropriate for the individual” [30] (p. 46). Apart from the main four types/specializations of AsTs (namely art therapy, music therapy, drama therapy, and dance/movement therapy), there is also an intermodal approach defined by Knill [31] called expressive arts therapies. Comparative research focusing on the unique contribution of every artistic media is needed for the further development of AsTs as a treatment intervention in the field of EDs.

EDs are present in both genders and in those with a more fluid understanding of gender, however to date EDs are more prevalent in the female population. To address gender aspects in the AsTs it is perhaps timely to mention the influence of feminist and post-feminist theories on the work with the female population in AsT practice. Approaches that lean towards exploring the social and cultural context of a person with an ED often have their roots in feminist theories as a way to gauge a broader perspective of issues that are directly linked to women. For instance Dokter [32] in her drama therapy (DT) practice identified that patients with an ED using DT as a treatment intervention are more likely to be female, and thus interventions/methods should reflect gender differences.

Compared to traditional verbal treatments, AsTs are described as non-verbal, creative, expressive, and experiential (action oriented). The creative process on a non-verbal level may lessen defense mechanisms, rationalization, intellectualization, and persuasion tendencies that are frequently used by patients with EDs when describing their symptoms and feelings on a verbal level [33]. The same defense mechanisms used by the patient to protect the self and provide a sense of control are often replicated in verbal psychotherapy. Reliance on rationalism, intellectualization, or arguments about the patients’ intimate relationships with food can slow the verbal psychotherapy process and stop patients from processing their emotions on a deeper level [34]. AsTs allow patients to work with other parts of their bodies through various methods and techniques such as playing music, painting, movement, or role-play. The shift to a more “right brain” creative approach which facilitates the psychotherapeutic use of music, art, movement, and drama opens the door for the patient to shift from explaining their feelings into actually feeling them. Alternative means of expression such as metaphors can build a bridge into a deep awareness that may be missed in a more verbal treatment. Plus the engagement of other body parts in the treatment of EDs can be crucial for patients’ reconnection with their bodies, as well as facilitating a better understanding and expression of emotions [10]. Although the argument for the use of creativity and non-verbal expression is both a valuable and practical resource that offers countless applications and interventions, there must also be a clear understanding of the possible risks. Physical activity as a compensation mechanism is a clear risk factor for patients who use exercise to lose weight, and therefore dance/movement therapy could be a more detrimental than helpful treatment path for the patient. As with all AsTs the therapeutic aim and intervention of the treatment must reflect the not just the ED but the individual.

As mentioned earlier, music therapy (MT), art therapy (AT), dance/movement therapy (D/MT), and drama therapy (DT) are the main four types of AsTs. Each approach has its own definition, specifics, methodology, and techniques. Since the careful description of each type of art therapy is required in more detail, the overview with specifications of each of these therapies can be found in Appendix C.

### 1.3. Justification of This Review Proposal and Its Objectives

There is a considerable lack of evidence-based research and outcome-focused studies in the field of AsTs and EDs [35]. While the AsTs have been clinically used as a treatment intervention in EDs, much of the published work in this area consists of case studies rather than the more robust method of randomized control trials [30,35].

The intention of the scoping review is to investigate and synthesize research evidence, aiming to map out the literature in the area of AsT interventions and their outcomes in their treatment of EDs. We will examine the scope, extent, and range of research activity undertaken in this area in order to determine the value and potential of AsTs as an intervention, and the outcomes of the treatment. Our objective is to be able to identity key concepts in the practice of AsTs and EDs, such as the therapeutic relationship, and categorize particular interventions and methods within each of the modalities: art therapy, music therapy, dance/movement therapy, and drama therapy. Although there is some primary research on AsTs and EDs, a search of Epistemonikos, Cochrane Reviews, JBI Evidence Synthesis, and Prospero showed no current or ongoing scoping review on this topic. The PCC format (P—participants/population, C—context, C—concept) was used to formulate two review questions [36]:

- What types of arts-related interventions are used in the treatment of persons with eating disorders?
- What therapeutic outcomes are addressed in research studies on persons with eating disorders related to arts therapies?

## 2. Materials and Methods

The proposed scoping review will be conducted in accordance with the Joanna Briggs Institute (JBI) methodology for scoping reviews [36] and according to PRISMA extension for scoping review [37] or its updates. Any deviation from this prospectively published protocol will be recorded and referred to in the full text of the scoping review.

Inclusion criteria:

- Participants: the review will consider studies that included persons of any age with eating disorders, including persons with various comorbidities such as other mental disorders (schizophrenia, schizotypal and delusional disorders, mood disorders, neurotic and stress-related disorders, disorders of adult personality and behavior, and mental disability).
- Concept: the review will consider any studies on AsTs, namely art therapy, music therapy, drama therapy, dance/movement therapy, as well as their combinations and expressive arts therapies. Excluded will be studies using arts for non-therapeutic objectives, e.g., for educational, personal, artistic, and other purposes.
- Context: the review will consider studies conducted in a broad geographical context or therapeutic setting without limitations.
- Types of sources: the review will consider all quantitative and qualitative research studies as well as systematic reviews, diploma thesis, and conference papers. Text, opinion papers, all types of non-systematic reviews, pre-conference abstracts, and bachelor theses will be excluded. There will be no time limit or language limit as long as the abstract of the paper is available in English.

The search databases will be: CINAHL Plus, EMBASE, MEDLINE (OvidSP), ProQuest Central, PsycINFO, PubMed, Scopus, and Web of Science. Sources of unpublished studies and grey literature will include Google Scholar, MedNar, clinical trials, and current controlled trials. An adapted search strategy will be used for grey literature in order to avoid an extremely high number of irrelevant results. An initial search was made in PubMed and PsycINFO (Appendix A). A manual search will be carried out in the selected books and chapters [33,34,38,39,40]. The reference list of all relevant studies will be screened for additional studies. The full search strategy for each database will be described in the full text of the scoping review.

Following the completion of the search, and to allow for the completion of a Preferred Reporting Items for Systematic Reviews and Meta-Analyses for Scoping Reviews (PRISMA-ScR) flow diagram [27], a full set of results will be exported from the sources into Zotero V5.0.85. Additional information, database name, export date, search terms used, number of results, grey literature sources searched, other search techniques, limitations, and duplicates will be recorded, alongside a customized field to provide comments. After the removal of duplicates, articles will then be selected in a two-step process by two reviewers working independently at each step:

- In step 1, two reviewers (MB and JK) will independently screen all the titles and abstracts returned for potential relevance. Each reviewer will assess the article against the inclusion and exclusion criteria. The proposed abstracts for full-text review will be compared between the two reviewers.
- Step 2 will be undertaken for each abstract selected. The full version of the article will be retrieved and imported into Zotero-5.0.85. Two reviewers (MB and JK) will independently undertake full-text analyses. Reasons for exclusion of full-text articles will be noted in Zotero-5.0.85 by each reviewer and provided as an appendix in the full review. The proposed full-text articles for the review will be compared between the two reviewers until a final set is agreed upon by both. The final set will be held in a Zotero-5.0.85 library. Where there is a disagreement between the two reviewers in either step, the final agreement will be sought by mutual consensus with input from a third reviewer (AM).

Data will be extracted from papers using a data extraction tool, which was developed by the authors (Appendix B: data extraction instrument). Two reviewers (MB and AM) will independently extract study data as per JBI scoping review methodology [36]. Data to be extracted from selected studies will include information about author and year, study design (quantitative studies will be described according to JBI levels of quantitative evidence), country, settings, population (number of participants, age, gender, comorbidities), methodology (data collection, analysis, description of experimental and control interventions), other therapeutic interventions (referred to in the study), type of AsTs, characteristics of arts-based interventions (materials, forms, methods/techniques, procedures/stages of therapy, length of treatment), and therapeutic outcomes related to AsTs.

## 3. Expected Results and Discussion

The extracted data will be presented in tabular form and as a narrative summary that aligns with the objective of the review. The table will report the following: author and year, study design (code according to JBI levels of evidence for quantitative studies), country, settings, population (number of participants, age, gender, presence of any comorbidities), methodology (measurements and any other methods used for data collection, methods of data analysis, and description of experimental and control interventions), other therapeutic interventions (referred to in the study), type of AsTs, and characteristics of arts-based interventions (therapeutic approach, materials, forms, methods/techniques, procedures/stages of therapy, length of treatment). This table may be further refined after data extraction and accompanied by graphic representations such as bar charts and diagrams. Any deviation from this protocol will be clearly detailed and justified.

In the proposed review we will compare arts/creative interventions according to different types of artistic media. This analysis may offer important information for AsT practitioners and their potential collaboration. The analysis of therapeutic outcomes related to AsTs will be important considering any potential future goals of AsT treatment of persons with EDs. The review will also offer a special comparison of therapeutic outcomes according to the type of AsTs. Furthermore, we will analyze the outcomes according to the type of research and study designs. We expect each type and design of empirical research to produce a special type of evidence that may concern the affectivity of treatment, the analysis of factors influencing the treatment, the patients’ lived experience with treatment, and the personal relevance of different kinds of treatment for every patient. Based on these findings (e.g., number of quantitative and qualitative studies), it will be possible to offer recommendations for future research that would motivate future advancements in the treatment of EDs through the invention of AsTs.

## 4. Conclusions

This is a protocol which is prospectively designed towards a scoping review of arts therapy interventions and their outcomes in the treatment of eating disorders. It can offer essential information for practitioners and researchers, and it will provide data for the development of future protocols of systematic reviews. Furthermore, it will offer a comparison of therapeutic interventions based on different arts’ medias used within each AsTs, which may potentially support the co-operation among arts therapists and stimulate a more systematic development of AsTs in the future.

## Appendix A. Search Formula and Preliminary Search in Databases

Search formula: eating disorder * OR anorexia OR bulimia OR anorexia nervosa OR bulimia nervosa OR binge eating disorder * (binge-eating disorder * or binge-eating syndrome * could be used as preferred term in the database) OR pica OR hyperorexia OR night eating syndrome * (night eating disorder * could be used as preferred term in the database) OR overeating OR orthorexia nervosa OR food intake disorder * and art therapy * OR art psychotherapy * OR music therapy * OR music medicine OR dramatherapy * OR drama therapy * OR psychodrama OR dance therapy * OR dance/ movement therapy * OR dance/movement psychotherapy * OR arts therapy * OR expressive therapy *.

## Appendix B

**Table A1:** Data extraction table.

Scoping Review Details	
Scoping review title:	Arts Therapies Interventions and their Outcomes in the Treatment of Eating Disorders
Review objective/s:	The aim of the prepared scoping review is to analyze the research studies on AsTs in the treatment of eating disorders.
Review question/s:	What types of arts-related interventions are used in the treatment of persons with eating disorders? What therapeutic outcomes are addressed in research studies on persons with eating disorders related to arts therapies?
Author	
Year	
Study design	
Country and settings	
Population	
Methodology	
Other therapeutic interventions	
Type of AsTs	
Characteristics of arts-based interventions	
Therapeutic outcomes related to AsTs	

## Appendix C

**Music therapy (MT)** uses music and its unique qualities, together with a strong therapeutic relationship, to address individual goals. The American Music Therapy Association [41] defines MT as the clinical and evidence-based use of music interventions to accomplish individualized goals within a therapeutic relationship by a credentialed professional who has completed an approved music therapy training program. The AMTA definition emphasizes the importance of evidence-based music interventions. MT can address and engage with specific characteristics exhibited by clients with EDs in different ways, depending on the music therapy approach used, and framework for understanding the illness.

When working with patients who have EDs, music therapists mostly use musical improvisation (with or without structure, role-plays), receptive methods (relaxation, guided imagery to music, song lyric discussion, responding to music through art, movement, or writing), and composing (songwriting) [10,42]. MT techniques can be applied in individual as well as in group settings, however there are arguments against group work for adolescents suffering from AN. Working with those patients separately prevents them from sharing strategies and behaviors supporting their restrictive practices and it also eliminates competitive factors within the group [10].

Playing music creates an opportunity for the client to be heard and acknowledged by the therapist without having to do anything about it. Music improvisation may enable clients to reconnect with other parts of themselves as well as with others, and may encourage the client to accept their current needs. This can be a challenging concept since patients with an ED often find it difficult to both experience and tolerate emotions. However, the benefits of a client expressing themselves through music can not only support the integration of thoughts and emotions, but build a base from which patients may learn to relax and create the capacity for self-soothing techniques [10].

**Art therapy (AT)** integrates psychotherapeutic techniques through a creative process which involves the use of painting, drawing, sculpturing, collage, and coloring. The American Art Therapy Association [43] characterizes art therapy as an approach to mental health that utilizes the process of creating art to improve mental, physical, and emotional wellness. AT is founded upon the belief that expression through artistic means has therapeutic value, and as clients create art they may interpret what they have made and how it makes them feel. It is through a process of exploring their art that clients can look for themes that might be affecting their thoughts, emotions, and behaviors [38].

Hinz [34] suggests the use of images in AT can bypass language-based defenses and go more directly to the core of the issue with an ED patient. Since most ED suffers have a highly developed rhetoric for their ED they are less likely to censor themselves in the artistic process and argue about what they see in the final image [34,44]. Both the art product and the creative process of the AT session give insights into understanding a person’s ED struggle. The art product/image can convey directly or indirectly how the patient represents themselves, their relationships, and their environment. A direct image of a patient´s family, for example, can be understood through the size, color, shape, and placement of figures on the page. Figures which are small and surrounded by space may indicate feeling lost and lonely. Images can also speak indirectly through metaphors which AT describes as the “unconscious (the part involved in creating), speaking to the conscious (the part involved in perceiving the finished product)” [34] (p. 15). As a result, the patient who consciously sees “safety” in the eating disorder may be faced with an unconscious self-revealing image that opposes the conscious rationalizing of the disease.

The process of creating art can be just as important as the image produced. Purposely chosen colors, shapes, and paper may provide an insight into the patient’s likes and dislikes. The exploration of certain combinations, whether complimentary or clashing, can show the patient in a clearer way than if they had been explaining likes and dislikes in verbal psychotherapy [34,44]. Therefore, the use of art allows the patient to participate in therapy and recovery without having to reveal themselves verbally earlier than is comfortable. In summary, AT expression is able to provide information from three sources: (1) the person, (2) the process of creating, and (3) the final product [34]. Additionally, the therapeutic art images chronicle the progression of the ED recovery which may otherwise be lost or elusive in verbal psychotherapy.

**Dance/movement therapy (D/MT)** recognizes body movement as an implicit form of expression. The Association for Dance Movement Psychotherapy (ADMP, UK) [45] defines the field as a “relational process in which client/s and therapist engage in an empathic creative process using body movement and dance to assist integration of emotional, cognitive, physical, social, and spiritual aspects of self”. As a treatment intervention, D/MT is practiced through individual or group sessions in wide range of health, education, social care, and private practice settings. The role of D/MT as an intervention in mental health continues to be explored and researched worldwide especially in the UK, USA, and Germany. Moreover, there is evidence-based research in the field of D/MT in the treatment of EDs [46,47] which indicates that D/MT can assist the patient with an ED through re-connection to feelings, improve mood states, and increased self-awareness [48]. The most recent findings from the Savidaki et al.’s [48] pilot study on the effects of dance/movement therapy on body image and alexithymia in eating disorders state that the participants of the study received the D/MT intervention positively, and appreciated the movement relationship with the therapist and the group. The ability D/MT has to address body-related issues means it has the potential to be more effective than the current verbal therapies when the crux of the psychological state is manifested in the body.

The body is the central battleground in EDs and as individuals focus on their thoughts, feelings are ignored. Ignoring feelings with processes that include and directly affect the body such as starvation, bingeing, and purging, amounts to burying the feelings through self-controlled physical sensation, the burial site being the body itself [49]. Subsequently D/MT can be seen as a promising, but potent treatment intervention, which may not be applicable for all patients. The dance/movement therapists’ role is to assist a patient with an ED to experience feelings and express them through their body language, with the aim of identifying connections between what they experience in the D/MT session and how it might reflect their lives [46,47]. For this reason, the therapeutic alliance is a crucial feature as dance/movement therapists use the signals from their own bodies to respond to the expression of the patient. Developing a therapeutic relationship involves the resonance (attunement) between patient and therapist and can lead to the exploration of deeper, unconscious feelings that have been buried in the body.

Kleinman [46], alongside Ressler [50], in her extensive work with D/MT and the recovery of patients with ED suggests there are three key concepts that underline the D/MT process as building blocks for recovery: rhythmic synchrony, kinesthetic awareness, and kinesthetic empathy. When a therapist is in tune or attuned to the rhythm of the patient, through rhythmic synchrony they sharpen their somatic awareness to their own feeling states as a way of understanding possible feelings present in the patient and how they respond to them, demonstrating kinesthetic awareness. From this information the therapist can make interventions based on the deeper understanding of the patients’ feelings. This capacity to experience the other is known as kinesthetic empathy, such as experiencing another’s body tension, and is significant to the conjoint journey that the patient and therapist share towards recovery. The intentions of D/MT is to encourage patients to gain a more realistic perception of their feelings around food and body image, by seeing themselves mirrored/reflected in the therapists’ movement [35].

**Drama therapy (DT)** is an embodied practice which the British Association of Dramatherapists’ [51] term as a “form of psychological therapy in which all of the performance arts are utilized within the therapeutic relationship”. Through the use of drama techniques such as role-play, storytelling, exploration of metaphors, empathy, distancing, witnessing, performance, and improvisation, drama therapy can support people by exploring painful and difficult life experiences in an indirect way [51,52,53].

A large percentage of the research on DT and EDs state that as an intervention DT often includes a level of psychodrama [32,54,55,56] in its application, especially in group sessions. Psychodrama founded by Moreno is a deep action method in which individuals and groups enact scenes from their lives, dreams, or fantasies in an effort to externalize unexpressed feelings, gain new insights, and practice new and more satisfying ways of being in the world. However DT literature does suggest that the experiential format of psychodrama, whereby the individual experiences the roles of protagonist, auxiliary ego, director, and observing group member, could uncover an underlying psychosis, or problem which may exacerbate an already fragile ED patient [32,57]. It seems the combination of psychodrama with the framework of DT and DT-focused methods has the potential to raise unsettling issues but provides structure and a clear technique for the expression of actions, emotions, and relationships. Since most patients with an ED may be anxious and easily frightened, it is clinically appropriate to provide predictability, structure, support, and clear explanations about each exercise [54].

The purpose of each method in DT is to better understand what the patient is trying to communicate and to offer a variety of channels in which to externalize the inner state, in order to assist psychological growth and behavioral change. As Rubin (in Brooke 2008) [54] says “dramatic enactment can create a bridge between human limitation and human aspirations, between who we are and who we want to become” (p. 174). Additionally, DT exercises according to Brooke [54] can evoke positive feelings and positive qualities in ED patients who have a rigidity about how they hold themselves and how they express themselves. For many patients the access to playfulness, spontaneity, resourcefulness, imagination, humor, and empathy represent the very qualities that have been frozen inside themselves [32,54].

There are six common DT concepts which lay down the psychological foundations of the DT intervention: transitional space, dramatic metaphor, embodiment, dramatic distancing, dramatic projection, and incorporation of additional modalities [51,52]. Each concept is aimed at concentrating either directly or indirectly on achieving integration of the mind and body, whilst encouraging the individual to see themselves as separate from their ED. The externalization and dramatic distancing assists patients in feeling they can safely analyze their attitude and behaviors within a safe transitional space, where anything can be imagined and therefore exist and potentially become reality [56].
